# Level of Agreement of Intraocular Lens Power Measurements Between a Swept-Source OCT Biometer and a Partial Coherence Interferometer

**DOI:** 10.3390/jcm14113903

**Published:** 2025-06-02

**Authors:** Eirini-Kanella Panagiotopoulou, Thomas Polychroniadis, Minas Bakirtzis, Ioannis Tsinopoulos, Nikolaos Ziakas, Georgios Labiris

**Affiliations:** 1 Department of Ophthalmology, University Hospital of Alexandroupolis, 68100 Dragana, Alexandroupolis, Greece; thompoly@med.duth.gr (T.P.); minas961@hotmail.com (M.B.); glampiri@med.duth.gr (G.L.); 2 Ocular Surgery, School of Medicine, Aristotle University of Thessaloniki, 54124 Thessaloniki, Greece; ioannis.tsinopoulos@gmail.com (I.T.); nikolasziakas@gmail.com (N.Z.); 3 2nd Department of Ophthalmology, School of Medicine, Aristotle University of Thessaloniki, Papageorgiou General Hospital, 56403 Thessaloniki, Greece

**Keywords:** Argos, IOLMaster 500, swept-source OCT, biometry, IOL power calculation

## Abstract

**Background/Objectives**: Swept-Source Optical Coherence Tomography (SS-OCT) is a novel optical biometry technology with limited published data on its reliability compared to the gold standard, partial coherence interferometry (PCI). This study aims to assess the agreement between an SS-OCT biometer (Argos) and a PCI device (IOLMaster 500) in terms of biometry values, intraocular lens (IOL) power calculation and mean prediction error (ME). **Methods**: In this prospective comparative study, axial length (AL), anterior chamber depth (ACD), flat (K1), steep (K2) and mean (Km) keratometry values, astigmatism power, J0, and J45 vector components, white-to-white distance (WTW), and IOL power calculations for nine IOL models using four formulas were compared in cataract patients. Refractive outcomes were assessed in eyes implanted with SN60WF and Panoptix IOLs, with ME calculated for each module and formula for both IOLs postoperatively. **Results**: This study included 133 eyes (mean age: 66.0 ± 10.95 years). Argos measured significantly higher ACD and steeper keratometry values than IOLMaster, albeit without significant differences in AL, astigmatism power, WTW, J0, and J45. Mean IOL power differences were within the clinically acceptable threshold (0.50 D), except for SN6ATx with Hoffer Q and Haigis, and Clareon with Haigis. For Panoptix and SN60WF, IOLMaster demonstrated a more hyperopic ME than Argos with SRK/T, Holladay 1, and Hoffer Q; however, this was without clinically significant differences. **Conclusions**: Argos and IOLMaster 500 presented differences in ACD, keratometry values, and IOL power calculation. However, both devices showed non-clinically significant differences in IOL power calculation and ME in the majority of formulas.

## 1. Introduction

Cataract surgery is the most common surgical procedure in medicine. After removing the cloudy crystalline lens, an intraocular lens (IOL) with an appropriately calculated IOL power is implanted in the eye aiming for distance vision without any residual refractive error. A variety of IOLs is available according to the desired postoperative refractive outcome. However, optimal refractive outcome depends highly on the accuracy of preoperative measurements and IOL power calculations [1].

One of the primary causes of unexpected residual refractive error in lens extraction surgery is incorrect IOL power selection and/or improper implantation [2]. For many decades, AL measurements of the eye were taken by ultrasound until Zeiss introduced PCI technology by launching IOLMaster 500 [3]. IOLMaster 500 is a non-contact optical biometer that implements PCI technology, which emits low-coherence laser light to obtain highly accurate and reproducible measurements. The AL is measured by a 780 nm laser diode infrared light. The ACD is measured as the distance between the lens and the cornea using lateral slit illumination. Finally, K readings are estimated by six points measured at a 2.5 mm zone on the anterior cornea [3,4].

IOLMaster 500 and, consequently, PCI technology has been the gold standard of biometry for years [5]. However, their main disadvantage is the limitation to measuring AL in patients with dense cataracts, posterior polar cataracts, and media opacities. This has been overcome by the new SS-OCT technology [6,7]. SS-OCT operates with a light source of 1060 nm wavelength, allowing for better tissue penetration and, thus, better image quality, succeeding in AL measurements of mature cataracts compared to conventional PCI biometry [7].

Argos is the latest SS-OCT optical biometer with integrated Verion image-guided surgery software, https://www.myalcon.com/international/professional/cataract-surgery/diagnostics/argos-biometer/ (Alcon Laboratories Inc., Fort Worth, TX, USA). It captures a 2D OCT image from the highest point of the cornea to the macula [5,7]. This OCT image can measure AL, ACD, lens thickness (LT), and central corneal thickness (CCT). Argos measures ALs from the corneal surface to the retinal pigmented epithelium by using refractive indexes that correspond to each tissue (standard refractive indices are 1.376 for cornea, 1.336 for aqueous and vitreous, and 1.410 for lens), so the final AL measurement is the total of four lengths in the four segments. Keratometry measurements are determined from the OCT image and a 2.2 mm-diameter ring of 16 projected LED reflections on the cornea, utilizing a corneal index of refraction of 1.3375 [7,8]. Another unique feature of Argos is the Enhanced Retinal Visualization (ERV) mode that amplifies the signal in the retinal area during measurements in mature cataracts [8].

The IOL power calculation is also based on the IOL power formulas designed to calculate the IOL power for the pseudophakic eye using mathematical models. They predict the refractive outcome of different IOL types and powers using biometric measurements, such as axial length (AL), and corneal power. The IOL power formulas can be classified according to their generation as first generation (SRK, SRK-I, Binkhorst formula), second generation (SRK-II, Holladay), third generation (SRK-T, Hoffer Q, Holladay-1), fourth generation (Holladay-2, Haigis), and fifth generation, also called the new-generation formulas (Barrett Universal II, Olsen, and the other new formulas) [9,10]. The popularity of the SRK/T, Holladay 1, and Hoffer Q formulas is attributed to their good results combined with the dependence on minimal data, i.e., corneal power and AL. On the contrary, the Haigis formula also needs the anterior chamber depth (ACD), while the Holladay 2 formula requires five additional parameters: the horizontal white-to-white (WTW), corneal diameter, lens thickness, refraction, and age.

All available formulas could be used in normal ocular anatomy. According to the relevant literature, short eyes show the largest variations in lens power calculation among the available formulas, while modern formulas tend to achieve the most reliable calculations regardless of ocular anatomy [10].

Within this context, the primary objective of the present study was to evaluate the level of agreement of the gold standard PCI biometer IOLMaster 500 (Carl Zeiss Meditec AG) and the recently introduced SS-OCT biometer Argos (Movu Inc., Santa Clara, CA, USA) in terms of AL, keratometry values, ACD, WTW, and IOL power calculation.

## 2. Materials and Methods

### 2.1. Setting

This was a prospective, comparative study. The study protocol adhered to the tenets of the Declaration of Helsinki and all participants provided written informed consent. The study protocol was approved by the Institutional Review Board of the University Hospital of Alexandroupolis (UHA). The study was conducted at the Ophthalmology Department in the UHA, Greece, between July 2023 and May 2024. The official registration number of the study is NCT05411341.

### 2.2. Participants

Patients who visited the outpatient service of UHA were enrolled on a consecutive-if-eligible basis. Eligibility criteria included age older than 18 and diagnosis of senile cataract, stage 2 to 4 of the Lens Opacities Classification System III (LOCS-III) grading scale. In contrast, exclusion criteria included diagnosis or evidence of dry eye disease, previous incisional eye surgery, and previous diagnosis of corneal disease.

### 2.3. Examination Technique—Data Collection

All study participants were evaluated with IOLMaster 500 and Argos in the same consistent way on the same day by the same operator. Specifically, according to the devices’ measurement protocols, Argos performed five AL, keratometry, astigmatism power, and WTW measurements, while IOLMaster 500 performed five AL and ACD measurements, three keratometry and astigmatism power measurements, and one WTW measurement. Both devices calculated the mean value for each parameter. The biometers were placed in the same examination room to ensure consistent light conditions. AL, keratometry (K1, K2, average K), ACD, astigmatism power, and WTW were obtained for the study and compared between the two modules. Test–retest reliability of Argos for all biometric parameters was evaluated in a 15-day time window by the same operator.

IOL power was calculated for the monofocal spherical SA60AT and aspherical SN60WF IOLs, Clareon CNA0T0, for the aspheric monofocal toric SN6ATx, for the trifocal diffractive Panoptix TFNT00 and Panoptix Toric TFNTx IOL, for the Vivity Extended Vision IOL DFT015 and Vivity Toric DFTx15 (Alcon Laboratories, Inc., Fort Worth, TX, USA), and for the aspheric monofocal Tecnis ZCB00 (Abbott Medical Optics, Santa Ana, CA, USA) using four formulas (SRK/T, Holladay 1, Hoffer Q, and Haigis). The IOL power for each IOL calculated with the four formulas was compared between IOLMaster 500 and Argos. IOLMaster 500 was set to a refractive index of 1.3375, the standard European setting, while Argos used refractive indexes that correspond to each tissue (standard refractive indexes are 1.375 [cornea], 1.336 [aqueous and vitreous] and 1.410 [lens]).

Moreover, refractive outcomes of eyes implanted with the SN60WF and Panoptix IOLs were measured 3 months after surgery using manifest refraction. Distance best-corrected visual acuity (BCVA) was assessed using the Democritus Digital Acuity and Reading Test (DDART) with letter charts. The test was displayed on a 55-inch smart TV with a 3840 × 2160 pixel (4 K) resolution, positioned at a distance of 3 m. Lighting conditions were standardized across all participants and verified using a portable lux meter (Extech EA30, Extech Instruments Corporation, Nashua, NH, USA). The examinees’ distance from the display was measured with a laser distance meter (Stanley TLM99s, Towson, MD, USA). The power of the implanted IOL was chosen based on IOLMaster 500, which is still considered the gold standard of biometry, and remains the clinical practice of our ophthalmology department. Actual postoperative spherical equivalent (SE) derived from manual refraction, and preoperative predicted SE according to both biometry modules were compared for each module and formula. The refractive target in the Panoptix eyes was emmetropia, while in the SN60WF eyes, was the first IOL power resulting in myopic residual refractive error. Prediction error was calculated for each module and formula for the two IOL models, for the IOL power selected for each participant, defined as the actual postoperative SE minus the preoperative predicted SE. In addition, the corresponding mean error (ME) was compared between the two modules, defined as the mean value of prediction error. Finally, the percentage of eyes with a ME within ±0.25, ±0.50, ±1.00 D, and ±2.00 D was calculated.

### 2.4. Statistical Analysis

According to an a priori power analysis, a power of 0.8 at the significance level of 0.05 for an effect size of 0.4 was achieved using 100 eyes. Taking into account the predetermined 20% dropout rate, 125 eyes were needed to recruit. The same analysis was performed for the supplementary analysis of two IOL models. A power of 0.8 at the significance level of 0.05 for an effect size of 0.4 was achieved using 42 eyes for each group. Efficacy measurement for the study group was attempted by calculation of the mean difference (D) between the two devices.

All data were collected in Excel (Microsoft Corp.) and analyzed statistically with SPSS, version 29.0.1.0 (SPSS Inc., IBM Corp. Chicago, IL, USA). The flat K readings (K1), steep K readings (K2), mean K readings (Km = [K1 + K2]/2), magnitude and flat axis of astigmatism, J0 and J45 vector components, ACD, WTW, and IOL powers were analyzed. The success rate of acquiring AL measurements with the two modules was examined.

The normality of the measured data was evaluated using the Shapiro–Wilk test. The comparison of the examined parameters between the two modules was assessed using the paired samples Student’s t-test and Wilcoxon signed ranks tests for normally and non-normally distributed data, respectively. The mean differences of measured parameters between the two devices and 95% limits of agreement were also presented using Bland–Altman plots. Intraclass Correlation Coefficients (ICCs) were used to evaluate the test–retest reliability of the biometric parameters measured with Argos. Jamovi statistical software, version 2.3.2.0, was used to generate Bland–Altman plots. *p*-values < 0.05 were defined as statistically significant.

Jackson cross-cylinder power vector components (J0 and J45) were calculated with the method described by Thibos and Horner [11,12]. Namely, the formulas J0 = (−[cylinder/2] cos [2 × axis]) and J45 = (−[cylinder/2] sin [2 × axis]) were used to determine the vector at Jackson cross-cylinder power at axis 0° and 90° (J0) and 45° and 135° (J45), respectively. After this conversion, the Cartesian coordinates were available for statistical comparison. The limits of agreement were defined as the mean ± 1.96 standard deviation (SD) of the differences.

## 3. Results

**Figure 1:**
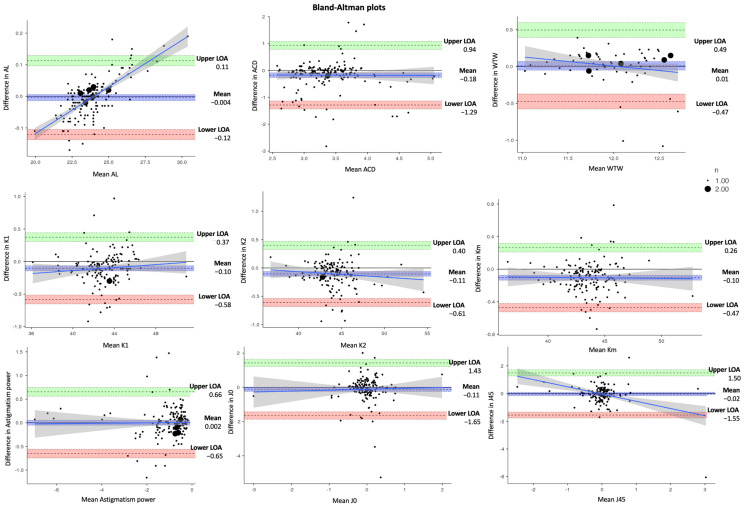
Bland–Altman plots for biometry parameters measured using IOLMaster and Argos (Difference: IOLMaster–Argos).

One hundred thirty-three eyes from 68 patients (41 men and 27 women) were included in this study. The mean age of the patients was 66 ± 10.95 years. Table 1 and Figure 1 show AL, ACD, the measurements of keratometry (K1, K2, and Km), astigmatism power, J0, and J45 vector components, and WTW. Compared with IOLMaster 500, Argos measured significantly higher ACD and steeper K1, K2, and Km values (*p* < 0.05), while no significant differences were observed in AL, J0, and J45 vectors, astigmatism power, and WTW (*p* > 0.05). As regards test–retest reliability, ICCs for all parameters were higher than 0.90, indicating excellent repeatability. The success rate of acquiring AL measurements with IOLMaster was 91.2% (6 cases with measurement failure), while Argos was 100% (0 cases with measurement failure).

Pearson correlation analysis was performed between all biometry values (measured with IOLMaster) and the corresponding differences between the two devices (inter-device differences) (IOLMaster–Argos) (Table 2). A significant positive correlation was observed in AL, ACD, astigmatism power, J0, and J45 (measured with IOLMaster). Indeed, the best level of agreement for AL was observed in AL equal to 23.6 mm, while in AL < 23.6 mm, Argos tended to measure longer AL, and in AL > 23.6 mm, tended to measure shorter AL.

**Figure 2:**
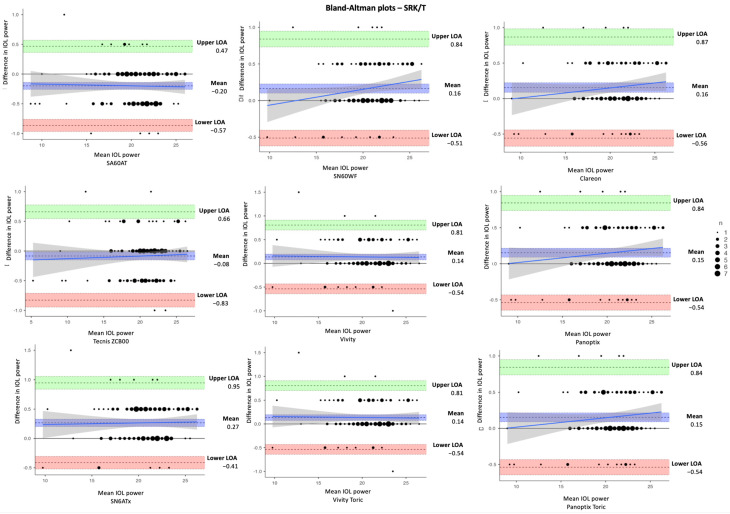
Bland–Altman plots for IOL power calculated with IOLMaster and Argos using SRK/T formula for the nine examined IOLs (IOLMaster–Argos).

**Figure 3:**
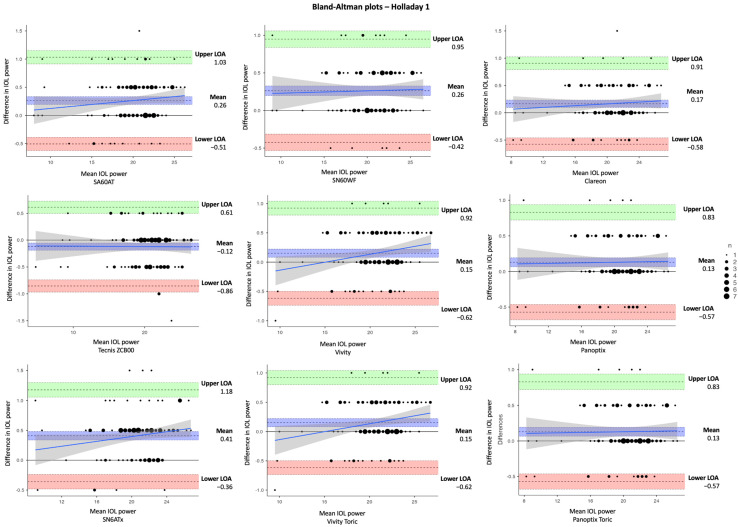
Bland–Altman plots for IOL power calculated with IOLMaster and Argos using Holladay 1 formula for the nine examined IOLs (IOLMaster–Argos).

**Figure 4:**
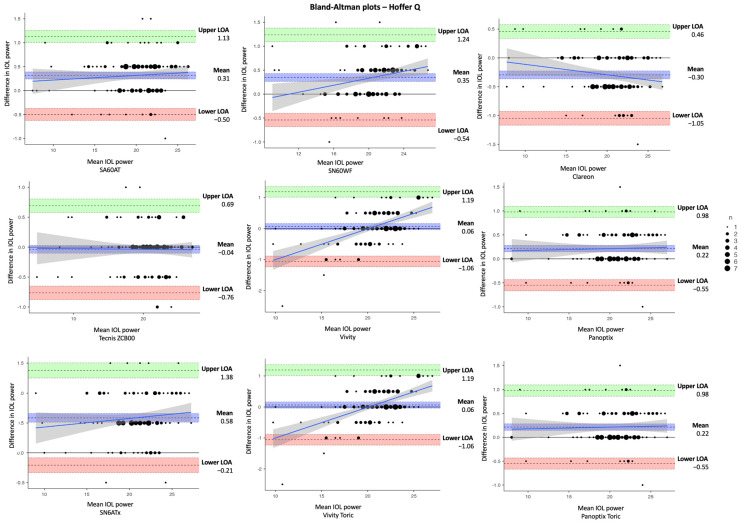
Bland–Altman plots for IOL power calculated with IOLMaster and Argos using Hoffer Q formula for the nine examined IOLs (IOLMaster–Argos).

**Figure 5:**
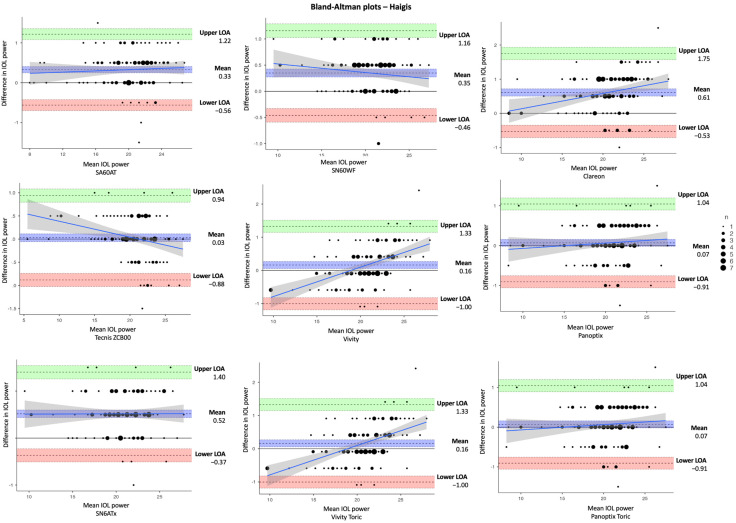
Bland–Altman plots for IOL power calculated with IOLMaster and Argos using Haigis formula for the nine examined IOLs (IOLMaster–Argos).

In Table 3, the estimated dioptric power of nine IOL models was compared using the SRK/T, Holladay 1, Hoffer Q, and Haigis formulas, respectively. The corresponding differences in IOL power between IOLMaster and Argos (IOLMaster–Argos) vs. the average in measured data are shown in Bland–Altman plots (Figure 2, Figure 3, Figure 4 and Figure 5). With the SRK/T and Holladay 1 formulas, IOLMaster 500 and Argos demonstrated a significant difference in the IOL power calculation of all IOL models. Moreover, the two devices showed a significant difference in the power of all IOL models apart from the Tecnis ZCB00 (*p* = 0.280), Vivity (*p* = 0.216), and Vivity Toric IOLs (*p* = 0.216) with the Hoffer Q formula, and Tecnis ZCB00 (*p* = 0.497), Panoptix (*p* = 0.129), and Panoptix Toric (*p* = 0.129) with the Haigis formula. All mean differences were lower than the clinically significant difference of 0.50 D apart from SN6ATx IOL with Hoffer Q (0.58 D) and Haigis (0.52 D), as well as Clareon IOL with Haigis (0.62 D).

Among the 133 participants, 64 were implanted with Panoptix IOLs and 69 with SN60WF IOLs, and both groups were included in the postoperative analysis. Seven patients were excluded because of postoperative complications. The ME for Panoptix and SN60WF for each formula between the two modules is compared in Table 4. For both IOLs, IOLMaster demonstrated higher (more hyperopic) ME than Argos with values ranging between 0.118 and 0.656 D, with SRK/T, Holladay 1, and Hoffer Q showing a non-clinically significant difference (0.118–0.276 D). Indeed, SRK/T for SN60WF power calculation showed a non-significant difference in ME (*p* > 0.05). Finally, the percentage of eyes with a ME within ±0.25 D, ±0.50 D, ±1.00 D, and ±2.00 D is shown in Table 5. Haigis formula appeared the lowest percentage of eyes with ME within ±0.25 D and ±0.50 D for both devices. Additionally, Argos showed a higher percentage of eyes with ME within ±0.50 D and ±1.00 D for both IOLs and all formulas compared to IOLMaster 500.

## 4. Discussion

The refractive status after eye surgery involving IOL implantation can heavily impact patients’ quality of life and activities of daily living [13]. Accurate and precise biometric measurements are crucial for achieving optimal refractive outcomes after cataract surgery [4]. In order to calculate the optical power of the IOL, several parameters are taken into consideration: K values, AL, ACD, and WTW, in combination with an appropriate calculation formula and an IOL constant [5,8]. AL is considered to be of utmost importance for the IOL power estimation [8,14].

The observed differences in biometric measurements between Argos and IOLMaster 500 provide important insights into how variations in device technology and measurement principles may influence preoperative planning. While both devices yielded consistent AL values overall, our subgroup analysis revealed a pattern: Argos tended to slightly overestimate AL in shorter eyes (<23.6 mm) and underestimate it in longer eyes (>23.6 mm). Although not statistically significant, this trend may have clinical relevance in cases with extreme values of axial length, where even small variations can impact refractive outcomes.

The significantly greater ACD and steeper keratometric values (K1, K2, Km) measured by Argos suggest that its measurement principles—particularly the use of a smaller (2.2 mm) central zone for keratometry—might account for the observed differences. Since the corneal curvature is typically steeper centrally, Argos’s design may inherently yield higher K readings compared to IOLMaster 500, which uses a slightly larger measurement zone (2.3–2.5 mm) [15]. This technical detail is critical for clinicians to consider, especially when switching between devices or interpreting longitudinal data from mixed biometry sources.

These steeper keratometric readings, combined with differences in ACD, contributed to lower IOL power predictions by Argos across most formulas and IOL models. However, when considering clinical relevance—defined as differences exceeding 0.50 diopters—only three IOL–formula combinations surpassed this threshold. This reinforces the idea that, although statistically significant differences exist between devices, they often fall within clinically acceptable margins.

Moreover, the significant positive correlations observed between IOLMaster measurements (AL, ACD, astigmatism power, J0, J45) and the inter-device differences support the notion that certain biometric parameters may be more sensitive to device-specific measurement techniques. Importantly, these correlations do not necessarily undermine the reliability of either device but rather highlight the need for consistency when selecting and using a biometry system in clinical practice.

Our findings align with those of Labiris et al. [16], who similarly reported steeper keratometry values using image-guided systems with smaller measurement zones. Such consistency across studies strengthens the external validity of our results and suggests that clinicians should account for measurement zone diameter when interpreting keratometry-based differences in IOL calculations.

Overall, while both Argos and IOLMaster 500 are accurate and reliable, they are not interchangeable without consideration of their methodological differences. For best practice, preoperative planning should remain consistent with the same device to minimize refractive surprises, particularly in patients with atypical biometric characteristics.

The comparison of ME for Panoptix and SN60WF for each formula between the two devices revealed a more hyperopic ME with IOLMaster compared to Argos in all formulas for both IOLs. However, the difference was non-statistically significant (<0.50 D) in all formulas except Haigis. In fact, SRK/T for SN60WF showed a non-significant difference in ME, ensuring the safe use of both devices for this formula and IOL. These results could be attributed to the higher keratometric values and lower IOL power measured with Argos, as well as the fact that IOL power, in this study, was chosen based on IOLMaster measurements, following our department’s clinical practice. Finally, Argos showed a higher percentage of eyes with ME within ±0.50 D and ±1.00 D for both IOLs and all formulas compared to IOLMaster 500, with Haigis showing the lowest percentage of eyes with ME within ±0.25 D, and ±0.50 D for both devices.

To our knowledge, limited literature data compare Argos and IOLMaster 500. Higashiyama et al. determined statistically significant, but not clinically significant differences between the AL measured with the two biometers in short- and long-AL subgroups, while no significant differences were found in intermediate-AL groups (*p* > 0.05) [14]. Similarly, Huang et al. proved a better success rate in AL measuring with Argos [17]. Hussaindeen et al. found no significant difference in AL and K measurements in children aged 11–17 [18]. Whang et al. showed no significant difference in the predictive accuracies of IOLMaster 500 and Argos optical biometers, except for in medium-long eyes, where the predictive accuracy of SS-OCT biometry was higher [19]. Shammas et al. compared AL and ACD measurements, and found a slight advantage of the SS-OCT in both parameters [20]. Finally, according to Yang et al., AL, ACD, and WTW significantly differed between the two devices, while there was no difference in K measurements. As measured with Argos, AL tended to be shorter in long eyes (AL > 26.0 mm) and longer in short eyes (AL < 22.5 mm). ACD was longer when measured with IOLMaster 500, while WTW was longer when measured with Argos. Argos demonstrated higher efficacy in conducting accurate measurements compared to IOLMaster 500 [5]. According to the aforementioned literature review, conflicting outcomes regarding inter-device biometry parameters comparison were observed.

Interestingly, Argos has been also well compared to other PCI biometers. In general, Argos showed good repeatability and no significant difference, or a slight superiority, in the final refractive outcome when compared with conventional PCI biometers [21,22,23,24,25,26]. Finally, Argos has been compared to other SS-OCT biometers. Romanek et al. and Sabatino et al. showed no difference in AL measurements between Argos and IOLMaster 700 [8,27]. On the contrary, Omoto et al. found significantly different AL measurements between Argos and IOLMaster 700 [1], and Savini et al. found that IOLMaster 700 overestimates AL calculation [28]. At the same time, Galzignato et al. [29] and Tamaoki et al. [30] showed a myopic error in long ALs (>25 mm) when measured with Argos, compared to IOLMaster 700–Eyestar 900 and IOLMaster 700–OA-2000, respectively.

The limitations of the present study should be acknowledged before interpreting our results. First, one study group was included in the present study with the same participants being examined with both biometry modules, and with IOL power being chosen based on the gold standard IOLMaster, instead of the inclusion of two study groups, each one examined with only one biometry module, and comparing the postoperative prediction error of each group. Second, the postoperative refractive outcome was analyzed only for two of the examined IOLs. This limited postoperative analysis, focusing on just one monofocal and one trifocal IOL, may reduce the generalizability of our findings across a broader spectrum of lens designs. We acknowledge that expanding the outcome analysis to include a wider range of IOL types and incorporating longer-term follow-up would provide more comprehensive insight into the comparative performance of the two devices. Moreover, although the success rate in AL measuring with the two devices was evaluated in the present study, participants with overmature, white or brunescent cataracts were excluded, probably resulting in the absence of failed AL measurements with Argos. Finally, in the present study, the Signal-to-Noise Ratio (SNR) calculated by IOLMaster 500 was not evaluated. Therefore, future studies could take SNR into consideration calculated by IOLMaster 500, and assess its correlation with the difference in AL measurements between the two biometers.

To our knowledge, this is the first attempt to evaluate and compare IOLMaster 500 with Argos in measuring astigmatism power, J0, and J45 vector measuring, as well as in IOL power calculation using four different formulas for nine different IOL models, and in assessing the postoperative spherical equivalent prediction error for a monofocal and a trifocal diffractive IOL.

## 5. Conclusions

In conclusion, this study demonstrated statistically significant differences between the IOLMaster 500 and Argos devices in several key biometric parameters, including ACD, K1, K2, Km values, and IOL power calculations. Argos consistently measured longer ACD and steeper keratometric values compared to IOLMaster 500, resulting in lower IOL power predictions across most formulas and IOL models. Despite these differences, the magnitude of deviation in IOL power was generally within clinically acceptable limits, indicating that both devices can be reliably used in daily clinical practice, provided consistency is maintained in preoperative planning.

Furthermore, IOLMaster 500 was associated with a more hyperopic mean error (ME) than Argos for both Panoptix and SN60WF IOLs. However, these differences were not clinically significant when using SRK/T, Holladay 1, and Hoffer Q formulas, and not statistically significant for SN60WF with SRK/T.

Based on these findings, our perspective is that both devices are suitable for routine biometry in cataract surgery, but clinicians should be aware of their inherent differences—particularly in AL extremes or when switching between devices. Future prospective studies may further explore the implications of device-specific measurements on postoperative outcomes and patient satisfaction.

## Figures and Tables

**Table 1:** Comparison of biometry parameters (IOLMaster–Argos).

Parameter	Mean ± SD		Difference		
	IOLMaster	Argos	Mean ± SE	95% CI	*p* Value
AL (mm)	23.84 ± 1.45	23.845 ± 1.40	−0.004 ± 0.005	−0.01, 0.005	0.385
ACD (mm)	3.30 ± 0.54	3.47 ± 0.54	−0.18 ± 0.04	−0.26, −0.09	<0.001 *
K1 (D)	42.97 ± 1.88	43.07 ± 1.86	−0.10 ± 0.02	−0.14, −0.06	<0.001 *
K2 (D)	44.08 ± 2.19	44.19 ± 2.21	−0.11 ± 0.02	−0.15, −0.06	<0.001 *
Km (D)	43.53 ± 1.96	43.63 ± 1.96	−0.10 ± 0.02	−0.14 ± 0.07	<0.001 *
Astigmatism power (D)	−1.114 ± 1.12	−1.116 ± 1.11	0.002 ± 0.03	−0.05, 0.06	0.943
J0 vector (D)	−0.08 ± 0.58	0.03 ± 0.56	−0.11 ± 0.06	−0.24, 0.01	0.068
J45 vector (D)	−0.02± 0.53	−0.04 ± 0.56	−0.02 ± 0.05	−0.08, 0.11	0.740
WTW (mm)	11.90 ± 0.39	11.95 ± 0.44	0.01 ± 0.03	−0.05, 0.07	0.729

* *p* < 0.05; ACD = Anterior Chamber Depth; AL = Axial Length; CI = Confidence Interval; D = Diopters; J0 = Jackson cross-cylinder power at axis 0° and 90°; J45 = Jackson cross-cylinder power at axis 45° and 135°; K1 = Flat keratometry; K2 = Steep keratometry; Km = Mean keratometry; SD = Standard Deviation; SE = Standard Error; WTW = White-to-white.

**Table 2:** Pearson’s correlation analysis between biometry values and inter-device differences.

Parameters	Pearson’s r	*p* Value
AL	0.715	<0.001 **
ACD	0.518	<0.001 **
WTW	0.118	0.331
K1	0.152	0.064
K2	−0.025	0.758
Km	0.034	0.681
Astigmatism power	0.162	0.048 *
J0	0.699	<0.001 **
J45	0.518	<0.001 **

* *p*-value < 0.05; ** *p*-value < 0.001; Inter-device differences: IOLMaster–Argos. ACD: Anterior Chamber Depth, AL: Axial Length, J0: Jackson cross-cylinder power at axis 0° and 90°, J45: Jackson cross-cylinder power at axis 45° and 135°, K1: Flat keratometry; K2: Steep keratometry, Km: Mean keratometry, WTW: White-to-white.

**Table 3:** Comparison of IOL power calculated with SRK/T, Holladay 1, Hoffer Q and Haigis formulas (IOLMaster–Argos).

IOL	Mean IOL Power ± SD (D)	Difference (IOLMaster–Argos) (D)			
	IOLMaster	Argos	Mean ± SE	95% CI	*p* Value
A. SRK/T					
SA60AT	20.2 ± 3.31	20.4 ± 3.32	−0.20 ± 0.03	−0.26, −0.14	<0.001 **
SN60WF	20.6 ± 3.10	20.4 ± 3.03	0.16 ± 0.03	0.10, 0.23	<0.001 **
Clareon (CNA0T0)	20.5 ± 3.51	20.3 ± 3.46	0.16 ± 0.03	0.09, 0.22	<0.001 **
Tecnis ZCB00	20.3 ± 3.65	20.4 ± 3.63	−0.08 ± 0.03	−0.15, −0.02	0.015 *
Vivity (DFT015)	20.8 ± 3.04	20.7 ± 3.05	0.14 ± 0.03	0.07, 0.20	<0.001 **
Panoptix (TFTN00)	20.5 ± 3.36	20.4 ± 3.32	0.15 ± 0.03	0.09, 0.21	<0.001 **
SN6ATx	20.8 ± 3.07	20.5 ± 3.06	0.27 ± 0.03	0.20, 0.33	<0.001 **
Vivity Toric (DFTx15)	20.8 ± 3.04	20.7 ± 3.05	0.14 ± 0.03	0.07, 0.20	<0.001 **
Panoptix Toric (TFNTx)	20.5 ± 3.36	20.4 ± 3.32	0.15 ± 0.03	0.09, 0.21	<0.001 **
B. Holladay 1					
SA60AT	20.1 ± 3.49	19.8 ± 3.43	0.26 ± 0.05	0.12, 0.31	<0.001 **
SN60WF	20.7 ± 3.35	20.2 ± 3.57	0.26 ± 0.05	0.13, 0.30	<0.001 **
Clareon (CNA0T0)	20.4 ± 3.57	20.3 ± 3.57	0.17 ± 0.05	0.03, 0.22	<0.001 **
Tecnis ZCB00	20.2 ± 3.93	20.3 ± 3.90	−0.12 ± 0.05	−0.28, −0.08	<0.001 **
Vivity (DFT015)	20.8 ± 3.28	20.6 ± 3.20	0.15 ± 0.05	0.08, 0.22	<0.001 **
Panoptix (TFTN00)	20.4 ± 3.56	20.3 ± 3.55	0.13 ± 0.03	0.07, 0.19	<0.001 **
SN6ATx	20.8 ± 3.35	20.4 ± 3.28	0.41 ± 0.04	0.34, 0.48	< 0.001 **
Vivity Toric (DFTx15)	20.8 ± 3.28	20.6 ± 3.20	0.15 ± 0.05	0.08, 0.22	< 0.001 **
Panoptix Toric (TFNTx)	20.4 ± 3.56	20.3 ± 3.55	0.13 ± 0.03	0.07, 0.19	< 0.001 **
C. Hoffer Q					
SA60AT	20.1 ±3.60	19.8 ±3.56	0.31 ± 0.04	0.24, 0.39	< 0.001 **
SN60WF	20.7 ± 3.41	20.3 ± 3.29	0.35 ± 0.04	0.27, 0.44	< 0.001 **
Clareon (CNA0T0)	20.5 ± 3.66	21.0 ± 3.72	−0.30 ± 0.03	−0.36, −0.23	< 0.001 **
Tecnis ZCB00	20.2 ± 3.96	20.3 ± 3.96	−0.04 ± 0.04	−0.10, 0.03	0.280
Vivity (DFT015)	20.8 ± 3.36	2075 ± 3.05	0.06 ± 0.05	−0.04, 0.16	0.216
Panoptix (TFTN00)	20.5 ± 3.66	20.3 ± 3.65	0.22 ± 0.03	0.17, 0.28	< 0.001 **
SN6ATx	21.0 ± 3.41	20.4 ± 3.36	0.58 ± 0.04	0.51, 0.66	< 0.001 **
Vivity Toric (DFTx15)	20.8 ± 3.36	2075 ± 3.05	0.06 ± 0.05	−0.04, 0.16	0.216
Panoptix Toric (TFNTx)	20.5 ± 3.66	20.3 ± 3.65	0.22 ± 0.03	0.17, 0.28	< 0.001 **
D. Haigis					
SA60AT	20.2 ± 3.54	19.9 ± 3.49	0.33 ± 0.04	0.25, 0.41	< 0.001 **
SN60WF	20.7 ± 3.27	20.4 ± 3.632	0.35 ± 0.04	0.27, 0.43	< 0.001 **
Clareon (CNA0T0)	20.9 ± 3.86	20.3 ± 3.69	0.61 ± 0.05	0.50, 0.72	< 0.001 **
Tecnis ZCB00	20.4 ± 3.77	20.4 ± 3.90	0.03 ± 0.04	−0.05, 0.11	0.497
Vivity (DFT015)	20.8 ± 3.59	20.6 ± 3.28	0.16 ± 0.05	0.06, 0.27	0.003 *
Panoptix (TFTN00)	20.4 ± 3.66	20.4 ± 3.61	0.07 ± 0.04	−0.02, 0.16	0.129
SN6ATx	21.0 ± 3.34	20.5 ± 3.33	0.52 ± 0.04	0.44, 0.60	< 0.001 **
Vivity Toric (DFTx15)	20.8 ± 3.59	20.6 ± 3.28	0.16 ± 0.05	0.06, 0.27	0.003 *
Panoptix Toric (TFNTx)	20.4 ± 3.66	20.4 ± 3.61	0.07 ± 0.04	−0.02, 0.16	0.129

* *p*-value < 0.05; ** *p*-value < 0.001; CI = Confidence Interval; D = Diopters; IOL = Intraocular Lens; SD = Standard Deviation; SE = Standard Error; SRK/T = Sanders–Retzlaff–Kraff/Theoretical.

**Table 4:** Comparison of the mean error for Panoptix and SN60WF IOLs for each formula between IOLMaster and Argos.

		Mean Error ± SD (D)		Difference (IOLMaster–Argos) (D)		
		IOLMaster	Argos	Mean ± SE	95% CI	*p*-Value
**Panoptix**						
	SRK/T	0.095 ± 0.136	−0.168 ± 0.173	0.263 ± 0.044	0.17, 0.36	<0.001 **
	Holladay 1	0.070 ± 0.164	−0.194 ± 0.111	0.264 ± 0.044	0.17, 0.36	<0.001 **
	Hoffer Q	0.050 ± 0.157	−0.210 ± 0.118	0.260 ± 0.033	0.19, 0.33	<0.001 **
	Haigis	0.330 ± 0.446	−0.188 ± 0.137	0.518 ± 0.081	0.35, 0.69	<0.001 **
**SN60WF**						
	SRK/T	0.290 ± 0.299	0.172 ± 0.208	0.118 ± 0.058	−0.01, 0.24	0.060
	Holladay 1	0.366 ± 0.324	0.162 ± 0.223	0.204 ± 0.066	0.06, 0.35	0.008 *
	Hoffer Q	0.440 ± 0.389	0.164 ± 0.272	0.276 ± 0.066	0.13, 0.41	<0.001 **
	Haigis	0.796 ± 0.572	0.140 ± 0.222	0.656 ± 0.100	0.44, 0.87	<0.001 **

* *p*-value < 0.05; ** *p*-value < 0.001; mean error: mean value of prediction error (actual postoperative SE—predicted SE). CI = confidence interval; D = diopters; SD = standard deviation; SE = standard error.

**Table 5:** Mean error for two IOL models and four formulas with IOLMaster 500 and Argos (percentage of eyes).

IOL/Formula	ME: ±0.25 D (%)		ME: ±0.50 D (%)		ME: ±1.00 D (%)		ME: ±2.00 D (%)	
	IOLMaster	Argos	IOLMaster	Argos	IOLMaster	Argos	IOLMaster	Argos
**Panoptix**								
SRK/T	87.5	87.5	100	100	100	100	100	100
Holladay 1	79.7	81.3	100	100	100	100	100	100
Hoffer Q	100	79.8	100	100	100	100	100	100
Haigis	59.4	62.5	67.2	98.4	81.3	100	100	100
**SN60WF**								
SRK/T	66.7	47.8	86.9	100	100	100	100	100
Holladay 1	46.4	40.6	81.2	100	100	100	100	100
Hoffer Q	27.5	33.3	79.7	100	81.2	100	100	100
Haigis	21.7	59.4	42.0	96.9	60.9	98.6	100	100

mean error: mean value of prediction error (actual postoperative SE—predicted SE). D = diopters; IOL = intraocular lens, ME = mean error.

## Data Availability

The data supporting the findings of this study are available from the corresponding author upon reasonable request. Due to ethical and privacy considerations, certain restrictions may apply to the availability of the data.

## Abbreviations

The following abbreviations are used in this manuscript:

ACD	Anterior chamber depth
AL	Axial length
CI	Confidence Interval
D	Diopters
DDART	Democritus Digital Visual Acuity Reading Test
IOL	Intraocular lens
J0	Jackson cross-cylinder power at axis 0° and 90°
J45	Jackson cross-cylinder power at axis 45° and 135°
K1	Flat keratometry value
K2	Steep keratometry value
Km	Mean keratometry value
ME	Mean Error
PCI	Partial Coherence Interferometry
SD	Standard Deviation
SE	Standard Error
SRK/T	Sanders-Retzlaff-Kraff/Theoretical
SS-OCT	Swept-Source Optical Coherence Tomography
WTW	White-to-white distance
